# Opioid agonist treatment take-home doses (‘carries’): Are current guidelines resulting in low treatment coverage among high-risk populations in Canada and the USA?

**DOI:** 10.1186/s12954-022-00671-z

**Published:** 2022-08-10

**Authors:** Cayley Russell, Shannon Lange, Fiona Kouyoumdjian, Amanda Butler, Farihah Ali

**Affiliations:** 1 Institute for Mental Health Policy Research, Centre for Addiction and Mental Health (CAMH), 33 Ursula Franklin St, Toronto, ON M5S 2S1 Canada; 2 Ontario Node, Canadian Research Initiative in Substance Misuse (CRISM), 33 Ursula Franklin St, ON M5S 2S1 Toronto, Canada; 3 Campbell Family Mental Health Research Institute, Centre for Addiction and Mental Health (CAMH), ON Toronto, Canada; 4 Department of Psychiatry, University of Toronto, Toronto, ON Canada; 5 Department of Family Medicine, McMaster University, Hamilton, ON Canada; 6 Centre for Urban Health Solutions, St. Michael’s Hospital, Toronto, ON Canada

**Keywords:** Carries, Corrections, High-risk populations, Opioids, Opioid use disorder, Opioid agonist treatment, Public health policy

## Abstract

Opioid agonist treatment (OAT) is the primary intervention for opioid use disorder (OUD) in Canada and the USA. Yet, a number of barriers contribute to sub-optimal treatment uptake and retention, including daily-supervised medication administration. Thus, clients are eventually granted access to take-home OAT doses (i.e., ‘carries’) to reduce this burden. However, this decision is based on physician discretion and whether patients can demonstrate stability in various life domains, many of which are inextricably linked to the social determinants of health (SDOH). Current Canadian and USA OAT carry guidance documents are not standardized and do not take the SDOH into consideration, resulting in the potential for inequitable access to OAT carries, which may be the case particularly among marginalized populations such as individuals with OUD who have been released from custody. This *perspective* article posits that current OAT guidelines contribute to inequities in access to OAT carries, and that these inequities likely result in disproportionately low coverage for OUD treatment among some high-risk groups, including individuals on release from incarceration in particular. Relevant impacts of COVID-19 and related policy changes are considered, and suggestions and recommendations to amend current OAT guidance documents are provided.

## Introduction

Opioid use disorder (OUD) is a complex illness, commonly characterized by chronic, lifelong relapse episodes and is associated with significantly high rates of morbidity and mortality [1]. Individuals with OUD often have poor health status and high levels of involvement in the criminal justice system, particularly due to the criminalization of substance use [2–6]. As per various clinical guidelines in Canada and the USA, as well as elsewhere, the gold standard for OUD treatment is opioid agonist treatment (OAT) [7–10]. OAT is evidence-based, safe and effective [1, 8, 11] and is associated with improvements in health care and addiction treatment engagement [9] and reductions in opioid use and related harms including all-cause mortality, overdose and suicide [12, 13]. These benefits underscore the vast health benefits and utility of OAT access as a key public health intervention. This may be particularly important within the context of the dual public health crises of the opioid overdose epidemic and the novel coronavirus 2019 (COVID-19) pandemic, where opioid-related harms have been substantially amplified [14–16].

Although OAT is available in both Canada and the USA, the way that it is prescribed and delivered to patients vastly differs, and in both countries, regulations and availability of providers also varies by state and/or province [17]. In the USA, methadone is highly regulated and only available through federally certified and accredited opioid treatment programs (OTPs) (i.e., general healthcare professionals cannot provide methadone), while buprenorphine is more widely available through family physicians; yet, only providers who apply for and receive a federal buprenorphine waiver can provide it [10, 18, 19]. In Canada, both methadone and buprenorphine are available in several clinical contexts including addiction program clinics, physician’s offices or pharmacies, and since 2018 providers are no longer required to obtain a federal exemption to prescribe methadone; however, physicians and nurse practitioners are still required and/or encouraged to take a training course and become certified in order to prescribe OAT in most provinces [9, 20, 21].

Beyond discrepancies in prescribing practices (i.e., stringent provider regulations, particularly in certain jurisdictions) which may limit or hinder OAT uptake, a number of barriers contribute to suboptimal OAT engagement and retention rates, including strict program requirements [17, 19]. For instance, individuals initiating OAT are typically required to visit a clinic daily for supervised medication administration, and to provide frequent (e.g., weekly) urine specimens [9, 10, 20, 22]. Many individuals deem these requirements as overly burdensome as the need to attend the clinic daily prevents them from achieving health and life goals and often drives decisions to discontinue or avoid OAT [23, 24]. In order to mitigate burdensome clinic visits, OAT guidelines in Canada and the USA state that after a number (typically two to three) of consecutive months of treatment, patients should be gradually provided ‘take-home’ or ‘unwitnessed’ OAT doses (often colloquially called ‘*carries,’* or *‘take-homes,’* with terminology varying by jurisdiction*)*, whereby the patient is provided OAT medication to self-administer without any supervision or requirement to attend a clinic [9, 10, 25]. In Canada and the USA, it typically takes upwards of eight months to a year for a patient to achieve a full week’s worth of OAT carries [10, 20]. The relevant guidelines in both countries lay out strict rules for how long a patient must remain in treatment and demonstrate stability before receiving carries; however, whether these rules are evidence-based is uncertain.

The decision to provide a patient access to OAT carries is ultimately made by their OAT clinician, who assesses whether the benefits of providing carries outweigh the potential risks, such as toxicity from dosing errors, medication non-adherence, and diversion [9]. The decision is often contingent on whether patients can demonstrate clinical, economic, social, and psychological ‘stability,’ which includes consistent attendance at clinic visits combined with reduced illicit drug use (and ideally abstinence from non-prescribed opioids), as well as positive family/social relationships and behaviors (e.g., no criminal activity), and the ability to safely store OAT medication [9, 10]. Thus, many of the criteria for OAT carry access therefore depend on the broader social determinants of health (SDOH). For instance, the necessity of access to a safe space to store medication is inextricably linked to housing and financial stability, and those who are unstably housed or have difficulties demonstrating ‘stability’ in various socioeconomic domains may be denied access. Moreover, individuals who have co-occurring physical and mental health diagnoses (who arguably have an even greater need for health care and increased flexibility and support) may not be able to demonstrate stability and be excluded from accessing OAT carries. These factors emphasize the intrinsic yet circular role that the SDOH play in relation to individuals being able to meet eligibility criteria to obtain OAT carries, whereby individuals may not be able to obtain OAT carries if they have co-occurring illnesses and/or do not have income or housing, yet are forced to visit an OAT clinic daily which may compete with their ability to receive support and/or obtain housing and employment.

The discretionary nature of clinician decisions, combined with a lack of standardization and any consideration for the SDOH across guidance documents contributes to the potential of inequitable access to OAT carries. This inequity may disproportionately impact specific populations, such as individuals with OUD who have been released from incarceration, individuals living in remote areas, individuals experiencing homelessness, and others who cannot easily demonstrate ‘stability.’ In this commentary, we aim to discuss how current Canadian and USA OAT guidelines may be resulting in low treatment coverage and inequitable burden among some populations, focusing on people with OUD who have been released from custody to illustrate this likelihood.

## Importance of access to OAT carries for individuals with OUD during community release

When individuals with OUD are released from incarceration into the community, they face heightened risks for adverse outcomes including relapse and overdose-related deaths, which are particularly common in the initial days and weeks post-release [26–33]. Providing OAT to these individuals pre-release combined with efforts to ensure continuity of care has been found to increase drug treatment entry and retention and reduce substance use, overdose, mortality, and recidivism [2, 34–41].

However, individuals face a variety of barriers to OAT access upon release that contribute to suboptimal treatment adherence and related consequences [42–46]. For instance, many are required to adhere to correctional release plans, which often include conditions such as obtaining employment and stable housing, undertaking correctional and/or addiction treatment programs, and attending frequent appointments with their probation/parole officers/other case managers [42, 47]. The literature has highlighted that during community reintegration, individuals have difficulties balancing conflicting priorities such as maintaining employment in conjunction with their OAT regimen, and often have to reschedule their workdays around daily OAT clinic visits and limited operational hours [43, 44, 46, 48]. This burden is heightened among those who reside in remote areas where they have to travel far distances in order to access a clinic [49].

To support community reintegration, access to take-home OAT carries is especially important for individuals post-release as it obviates daily clinic visit requirements, allows the freedom and flexibility to take medication at a convenient time, and eases the burden of having to balance competing priorities such as visits to OAT clinics and other release requirements. However, it is also likely that individuals on release experience inequitable access to OAT carries considering they already face distinct challenges in regard to the SDOH, many of which are known to contribute to negative health outcomes (e.g., employment, income, housing, etc.), yet are required to demonstrate ‘stability’ [50–54]. As a key example, upon release, some individuals are given a residency restriction which requires them to reside within a community-based residential facility (i.e., a ‘halfway house’), where they live temporarily while they reintegrate into the community [55]. This can intrinsically act as a barrier to obtaining OAT carries since, by nature, halfway houses are considered temporary housing as individuals only remain there for short periods of time (typically a few months up to a year) while they finish the remainder of their sentence under community supervision [55, 56]. In these facilities, individuals live in a group environment under strict rules where they must abide by stringent release conditions [56]. During this period, OAT clinicians may be hesitant to grant an individual OAT carries for a number of reasons, including the risk for medication diversion and the temporary (i.e., unstable) nature of their living environment.

Furthermore, halfway houses and staff are often poorly equipped to facilitate effective OUD treatment [57]. Access to medications is commonly constrained by structural and organizational factors, including restrictive policies that limit the circumstances under which an individual can become eligible to receive carries while residing on-site. For example, contracts between correctional institutions and halfway houses set out detailed requirements for the storage of medications [55]. In regard to OAT carries, some contracts indicate that carries will only be temporarily kept on-site during exceptional circumstances (e.g., when the clinic is closed) [58], forcing individuals residing at halfway houses to visit an OAT clinic even after they have shown clinical stability and would otherwise likely be granted access, if their living situation were different. Results from a longitudinal mixed-methods study examining experiences with OAT among individuals recently released from federal incarceration in Canada indicated that many individuals who were residing at halfway houses during their community reintegration period were prohibited from storing OAT carries on-site [59]. For instance, one individual stated *“I don’t know why, but we can’t have [OAT carries] here [at the halfway house]…[I’d be eligible for carries] if I moved out tomorrow into my own spot…so it’s like being married to a drug store…I get my [OAT] at [clinic name], which takes me 45 min from here to get there on busses…and it’s really frustrating…it’d just be great if I didn’t have to go there every day.”* These findings underscore the potential for current OAT policies and related structural barriers to impede access to OAT medication and hinder patient-centered care among individuals with OUD on release from incarceration and, thus, likely result in low treatment coverage.

### COVID-19: special considerations and circumstances

The potential for individuals with OUD who have been released from incarceration to experience disproportionate access to OAT carries is even more concerning in light of COVID-19. Since the onset of the pandemic, OUD and related harms (i.e., opioid toxicity overdose deaths) have reached unprecedented heights [15, 16, 60, 61]. This situation underscores the need for increased access to OAT and highlights the specific utility of OAT carries during this time, which allows individuals the ability to stay home, physically distance, and mitigate the risk of exposure to the virus [62, 63]. Carries furthermore reduce access issues resulting from limited service capacity, scope, and hours, due to the implementation of required public health measures undertaken at many of the health and social services people who use drugs (PWUD) commonly use [64].

Recognizing this, amendments have been made to existent Canadian and USA OAT policies in order to facilitate access to and continuity of care for individuals on OAT during the pandemic [65–69]. The updated guidelines encourage a number of strategies to reduce the frequency of OAT clinic visits such as decreasing urine drug testing requirements and expanding virtual care and access to OAT carries, i.e., increasing the number of carries provided and loosening eligibility criteria [21]. Preliminary data highlight that those who were able to access OAT carries due to the revised policies benefitted from this change, particularly as access to carries permitted them to stabilize their routines, physically distance to reduce the risk of virus transmission, and subsequently decrease their substance use [18, 64, 68, 70–72]. For instance, in a US-based qualitative study emphasizing patient’s perspectives, many individuals acknowledged significant benefits of receiving carries during COVID-19 including experiencing feelings of normalcy and stability, improvements to recovery support, and increased time with family and work—benefits which increased their self-confidence and commitment to OAT adherence [73]. Early Canadian data further indicate that most people who received additional carries were no more likely to experience adverse outcomes (e.g., self-reported opioid overdoses, emergency department visits, etc.) than those who did not receive carries [74, 75]. However, most clinicians reported that they prescribed carries to patients only when they felt sure of the patient’s social and housing stability, which means that the perpetuation of inequitable access to OAT carries still exists for some high-risk populations, even during a period of loosened OAT carry prescription policies. Furthermore, while pre-COVID-19 OAT guidance included considerations for incarcerated individuals [9, 10], none of the updated guidance documents explicitly included this high-risk population in their amendments.

Whether or not the amendments to OAT guidelines led to easier access to OAT carries for individuals on community release remains unknown. Future research should evaluate potential inequities in access to OAT carries and related outcomes experienced by subpopulations, such as people on release from incarceration. Access and outcomes research could be used to inform the evidence-base for the equitable provision of OAT carries, including in the context of emergencies, and thus inform guidelines and policies which aim to address issues related to the SDOH and support increased treatment coverage. These policies would likely lead to better population health status for people with OUD, and particularly for those that have traditionally failed to meet OAT carry eligibility criteria due to their unique circumstances, including in relation to the SDOH.

## Conclusion

Overall, available data indicate that access to OAT carries in Canada and the USA is inequitable, including during the COVID-19 pandemic when guidelines regarding the provision of OAT care have been amended to increase access. We posit that OAT guidelines have contributed to inequities in access to OAT carries, and that this is likely to result in disproportionately low coverage for OUD treatment in some high-risk populations. This includes individuals with OUD released from incarceration who are at a heightened risk of experiencing treatment barriers and thus have a greater need for access to OAT carries. This inequity poses risks to those attempting to manage their substance use, maintain treatment, adhere to conditions of release, and effectively reintegrate into society.

Current OAT guidelines should be amended and standardized to explicitly include provisions for granting high-risk populations (e.g., individuals on community release) access to OAT carries, and to take the SDOH into better consideration when coming to these decisions, while also not negating potential risks (e.g., diversion) and associated harms among these populations. Additionally, alternative pharmacotherapies such as extended-release injectable/implantable OAT formulations that require less frequent clinic visits, or the use of telehealth to monitor at-home OAT ingestion, as well as novel OAT carry storage options that use biometrics to allow access to stored medications and reduce the risk of medication diversion (e.g., MySafe) [76] are all avenues that should be explored. Explicitly, correctional settings should minimally ensure OAT continuity during community release periods through the use of extended OAT prescriptions that allow for carries, and halfway houses should allow the storage of OAT carries on-site. These policy recommendations could help alleviate many of the issues discussed and would help ensure that individuals can work toward community reintegration and reach their health and life goals, and are not inequitably denied access to treatment due to criteria that do not align with their unique health status and life circumstances. This would ultimately increase OAT treatment coverage for high-risk populations, which may be particularly important given the current dual public health crises.

## Data Availability

Not applicable.

## Abbreviations

COVID-19: Novel coronavirus 2019
OUD: Opioid use disorder
OAT: Opioid agonist treatment
SDOH: Social determinants of health
PWUD: People who use drugs
